# Viral tag and grow: a scalable approach to capture and characterize infectious virus–host pairs

**DOI:** 10.1038/s43705-022-00093-9

**Published:** 2022-02-01

**Authors:** Ho Bin Jang, Lauren Chittick, Yueh-Fen Li, Olivier Zablocki, Courtney M. Sanderson, Alfonso Carrillo, Ger van den Engh, Matthew B. Sullivan

**Affiliations:** 1 Department of Microbiology, The Ohio State University, Columbus, OH USA; 2 Marine Cytometry Inc, Concrete, WA USA; 3 Department of Civil, Environmental and Geodetic Engineering, The Ohio State University, Columbus, OH USA; 4 Center of Microbiome Science, The Ohio State University, Columbus, OH USA

**Keywords:** Biological techniques, Population dynamics, Bacteriophages

## Abstract

Viral metagenomics (viromics) has reshaped our understanding of DNA viral diversity, ecology, and evolution across Earth’s ecosystems. However, viromics now needs approaches to link newly discovered viruses to their host cells and characterize them at scale. This study adapts one such method, sequencing-enabled viral tagging (VT), to establish “Viral Tag and Grow” (VT + Grow) to rapidly capture and characterize viruses that infect a cultivated target bacterium, *Pseudoalteromonas*. First, baseline cytometric and microscopy data improved understanding of how infection conditions and host physiology impact populations in VT flow cytograms. Next, we extensively evaluated “and grow” capability to assess where VT signals reflect adsorption alone or wholly successful infections that lead to lysis. Third, we applied VT + Grow to a clonal virus stock, which, coupled to traditional plaque assays, revealed significant variability in burst size—findings that hint at a viral “individuality” parallel to the microbial phenotypic heterogeneity literature. Finally, we established a live protocol for public comment and improvement via protocols.io to maximally empower the research community. Together these efforts provide a robust foundation for VT researchers, and establish VT + Grow as a promising scalable technology to capture and characterize viruses from mixed community source samples that infect cultivable bacteria.

## Introduction

Viruses that infect bacteria (phages) have been studied for over a century as model systems to establish the fundamentals of molecular biology and genetics, as well as their value as reagents or tools in these disciplines [1–5]. For example, studying phage-host model systems from medically relevant genera (e.g., *E.coli* [1, 4], *Mycobacterium* [2], *Pseudomonas* [3]), and key food production genera (e.g., *Lactococci* [5]) has led to seminal advances including the elucidation of restriction-modification systems, the realization that DNA was the carrier molecule of genetic inheritance, phage lambda as a cloning vector, first genomes sequenced, phage-derived enzymes central to molecular biology practices, CRISPR-based genome editing, and many more. Thus a first and second “age of phage”, respectively, revealed their promise as antagonists to pathogens (i.e., phage therapy [6]) and the mechanistic underpinnings of how cells and viruses work in the laboratory.

We are now well into the third age of phage [7] whereby ecologists seek to better map the “virosphere” (the diversity of virus sequence space that exists in nature) and elucidate the roles viruses play in complex communities. In this age, microbiologists have revealed that microbes support healthy ecosystems functioning in natural systems (e.g., global biogeochemical processes [8], climate change feedback loops [9], and now human systems [10]), there is increasing awareness that viruses that infect these microbes are also abundant and impactful. For example, in the oceans, the millions to hundreds of millions of viruses per milliliter of seawater are credited with killing approximately one in three cells per day [11], moving 10^24^ genes per year globally [12], and metabolically reprogramming infected cells (as “virocells” [13])—which together drastically alter the ecosystem outputs of marine microbes. Parallel findings are starting to emerge from soils [14, 15], extreme environments [16–18], and mammalian respiratory [19] and digestive systems [20–22]. Further, sequence-based viral metagenomic survey approaches have transformed our understanding of viral diversity, both by illuminating under-explored regions of the virosphere, even hundreds of thousands of viruses at a time [23], and by utilizing such genome-scale datasets to establish systematic definitions of viral “species” [23–25] and “genera” [26, 27]. Recently, buoyed with hybrid short- and long-read sequencing technologies, population-based ecogenomic analyses are expanding to applying measurements of selection of natural virus communities [28]. Thus, the modern viral ecogenomic toolkit [29] is capable of resolving multiple levels of diversity over scales as vast as the global oceans.

As the ecological importance for microbial communities is revealed, there is increasing interest in manipulating the microbiota [30] and its “theater of activity”—the Microbiome [31]—that is expected to revolutionize personalized medicine [32, 33], agriculture [34], food production [35, 36], and numerous other processes that microbiomes impact. A bottleneck to such efforts is that resolving biological interactions between viruses and their microbial hosts and characterizing virus phenotypes beyond genomes in complex communities is not keeping up with the blistering pace of virus discovery. There has been significant effort in the latter area with several methods developed to experimentally determine virus–host pairs at multiple scales using flow cytometry [37–39], droplets [40, 41], or electrophoresis [42]. While each key advances, these efforts are limited by (i) high biomass requirements (e.g., high virus titer) [37], (ii) inferring only hosts with viral marker genes [40], and (iii) assuming that adsorption equals infection [38, 39, 42].

The specifics of these limitations are as follows. First, droplet-based PCR combined with gene fusion, epicPCR, enabled in situ prediction of virus–host pairings in natural aquatic microbial communities [40]. However, it suffers from the requirement of a single viral marker gene (e.g., ribonucleotide reductase) and does not provide in vivo biological data on virus–host interaction dynamics. Second, AdsorpSeq [42] is a recently developed tool to identify phages for target bacteria through the gel-based separation of bacterial membrane-bound phages. Although AdsorpSeq successfully detected 26 new phage-host pairs from sewage samples, researchers cannot differentiate whether bound phages lead to productive lytic infections [42]. Similarly, viral tagging (“VT”) [38, 39]—fluorescently labeling DNA of “wild” virus particles, mixing these with a target, cultivable host bacterium, and assessing the population of cells via flow cytometry for a fluorescent shift attributed to the “viral tagging”—has helped improve our understanding of cyanophage and *Pseudoalteromonas* phage biology [38, 39] and deduce virus–host pairings in the human gut [43]. However, like AdsorbSeq, VT suffers from the criticism that it remains unknown whether virus–host pairs detected represent productive lytic infections [44, 45]. For both methods, this could be problematic as there are numerous known mechanisms whereby adsorption does not equal infection, including homoimmunity due to lysogeny [42, 46], reversible and/or nonspecific binding [39], or post-adsorption cellular defenses [47]. Further, broad adoption of VT is hindered by the lack of robust methods foundation and the diverse expertise (e.g., flow cytometry, sequencing, phage biology) needed for successful application.

Here we sought to resolve the latter issue via deep benchmarking of VT methods and, once robust, to resolve the former issue via establishing a plate-based “and grow” variant to push these approaches further. To maximize impact, we also established a community feedback forum via a “live protocol” at protocols.io, which we hope will help galvanize a robust user community and alert researchers broadly of some of the more challenging aspects of these methods.

## Results and discussion

### Improving our understanding of “viral tagging” flow cytometric signals

**Fig. 1:**
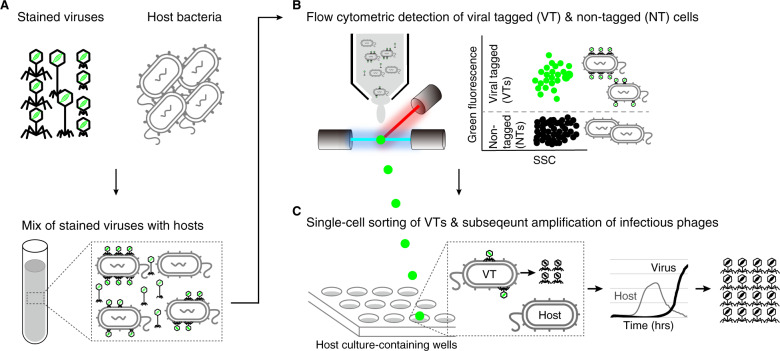
Overview of viral tagging, and the variant developed here—viral tag and grow. **A** Viruses are labeled with a green fluorescent dye and then mixed with potential host bacteria. **B** Fluorescence detection of individual cells with fluorescently-labeled viruses (FLVs) by flow cytometer. The flow cytometry plot (side scatter or forward scatter versus green fluorescence) shows the expected locations of FLV-tagged (VTs) and nontagged cells (NTs), which are flow-cytometrically green positive and negative, respectively. **C** Single-cell sorting of VTs is followed by subsequent amplification of infectious viruses. Single VTs are sorted into a 96-well plate that contains host culture. Culture growth is monitored by measuring optical density (OD) over time. A decrease in the OD curve from VT-containing wells (relative to the phage-negative control) indicates cell lysis by progeny viruses produced from a single isolated VT cell.

VT is a deceptively simple idea whereby a mixture of natural viruses are labeled with a DNA-binding fluorescent dye and ‘bait’ hosts infected by these stained viruses can be detected with flow cytometry via the fluorescent shift of “viral-tagged cells” [38, 39] (Fig. 1A, B). These viral-tagged cells can then be sorted, and the viral DNA separated using isotopic fractionation (the DNA of the cultured host is pre-labeled with “heavy” DNA) to access the metagenomes of the viruses that were experimentally determined to have infected these cell types. However, in practice, VT has been only minimally adopted by the community [43], presumably because it requires costly equipment (a high-performance flow sorter) and diverse technical expertise (flow cytometry, phage biology, and bioinformatics), while lacking sufficient benchmarking. To the latter, we sought to use a cultured phage-host model system (*Pseudoalteromonas* strain H71, hereafter H71, and its specific myophage PSA-HM1, hereafter HM1) to systematically assess the impact of various multiplicities of infection (MOIs; the ratio of the number of virus particles to the number of target cells, [48]) on the resultant VT signals. Further, we sought to augment VT to add an “and grow” capability whereby scalable single-virus cultivation, characterization, and sequencing could be enabled (Fig. 1C).

To gain a better understanding of the biology behind VT signatures, we examined how H71 interacts with HM1, a phage specific for this host, and HS8, a phage that does not adsorb to this host – both assayed via flow cytometry and microscopy (for details, see Methods and online protocol, https://www.protocols.io/view/viral-tagging-and-grow-a-scalable-approach-to-captbwutpewn?form=MY01SV&OCID=MY01SV). Briefly, phages were stained with SybrGold (fluoresces green upon blue-light excitation) and for microscopy, H71 cells were stained with DAPI (fluoresces blue upon blue-light excitation, 4′,6-diamidino-2-phenylindole), as previously described [39, 49]. Replicate cultures of stained cells were then mixed with fluorescently-labeled phages (either HM1 or HS8 in each treatment) at infective MOIs = 1, 2, and 4, then these infections were incubated for 10 min, and processed (centrifuged and resuspended; see Methods for details) three times to remove free phages (see Methods for details). For microscopy, the relative fraction of virus-tagged (VTs) and nontagged cells (NTs) was measured from the available cells up to ~500 cells for each sample. For flow cytometry, cell detection was optimized to minimize background noise [50], and negative controls consisted of stained and washed sheath buffer and filtered Q water samples, as previously described [39].

**Fig. 2:**
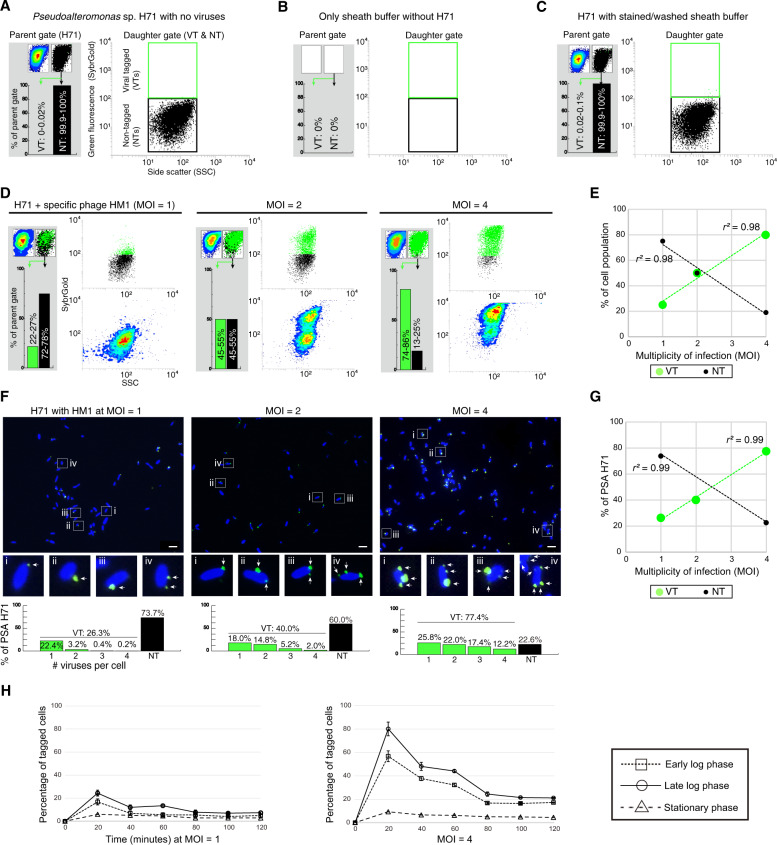
Flow cytometric and microscopic analyses of *Pseudoalteromonas*-phage associations. **A** Hierarchical gating for detection of *Pseudoalteromonas* strain H71 (hereafter, H71) and its subpopulations of viral tagged (VTs) and nontagged cells (NTs). A parent gate was drawn on H71 cells using FSC vs. SSC (Fig. S1) and represented in two types of contour and dot plots (left and right in the top of the gray box, respectively). From this gate, green-positive (VT) and -negative (NT) populations were sub-gated in the green fluorescence vs. SSC (right, dot plot) and quantified as percentage fractions of a parent population (bar charts in the gray box). **B**, **C** Flow cytometric plots of sheath buffer only (**B**) and stained/washed sheath buffer without phages (**C**) (see Methods and Fig. S1). **D** Flow cytometric detections for H71 cells (~10^6^/ml) that were incubated with fluorescently-labeled specific phage HM1 at MOIs of 1, 2, and 4, respectively (from left to right). **E** Linear regression relationships between the MOIs (x-axis) and the percentages (Y-axis) of flow cytometric VT (green) and NT (black) populations for phage HM1 at MOIs of 1, 2, and 4, respectively. R-square values are represented. **F** DAPI (4′,6-diamidino-2-phenylindole, blue)-stained H71 cells were mixed with fluorescent phages HM1 (SybrGold, green) at MOIs of 1, 2, and 4, respectively (Methods for details). Above, the merged images of phage-host mixtures (Additional images are shown in Figs. S4–7). Below, an enlarged view of four regions selected from the above images. Interpretations of virus-tagged cells, nontagged cells, and “free” viruses are represented in the results and discussion and methods, respectively. Arrows point to phages found on the margin of bacterial cells. Scale bar, 2 µm. Microscopic observations for nonspecific phage HS8-H71 are shown in Fig. S8. **G** Correlation between the MOI (x-axis) and the microscopic fractions (y-axis) of VTs (green) and NTs (black) for phage HM1 at MOIs of 1, 2, and 4, respectively. R-square value is shown. **H** Impact of cell physiology on viral tagging signals. H71 cells (~10^6^/ml) in the early log, late log, and stationary phase were infected by phage HM1 at MOIs of 1 (Left) and 4 (Right), respectively. Percentages of tagged populations were measured at the time point after fluorescently-labeled HM1 were inoculated for 20 min at various MOIs followed by centrifugation and resuspension to remove free viruses (see Methods for details). Each test was done in duplicate (error bars show standard deviations).

Overall, the resulting VT experiments were robust and informative. First, our cell-only optimizations resulted in controls that were impeccably clean (see representative cytograms and gating counts in Fig. 2A–C and  Supplementary Fig. S1). Second, in “virus addition” treatments, the resultant VT signal was distinct for specific (HM1) versus nonspecific (HS8) phages. Specifically, adding HM1 at MOIs = 1, 2, and 4 corresponded to VT population shifts of an average of 25%, 50%, and 80%, respectively, while NT populations proportionally decreased (Fig. 2D, E, linear regression *r*^*2*^ = 0.98). In contrast, for all tested MOIs of the nonspecific HS8 phage, the shifted populations were negligible (range: ~1.0–1.9%) and uncorrelated (Supplementary Fig. S2A, B; *r*^*2*^ = 0.14).

Despite observing a strong linear correlation between MOI and %VT for HM1, it was surprising that even at high MOIs = 1, 2, and 4, the resultant population shifts were 1.2- to 2.5-fold less than expected from theory alone based on Poisson distribution (see Supplementary Fig. S3). To investigate this, we used microscopy to inspect for virus clumping, positioning relative to cell surfaces, and background noise. These results revealed spot-like green signals of various sizes outside of host cells, which we interpreted as free viruses, and this was true even (a) at these higher MOIs, and (b) despite centrifugation to remove free viruses following incubation (see Methods; Fig. 2F and  Supplementary Figs. S4–7). We suspect these unincorporated SYBR-stained particles are viral aggregates, possibly due to host cell parts and/or debris in the lysate [51–53] or tangling of phage tails [54]. Prior work has shown that these and other mechanisms that decrease the accessibility of viral particles to host receptors could reduce observed infectious particles [48].

Our third key observation in these experiments rested with an improved understanding of the ‘signal shift’ between VT and NT populations in the flow cytogram across varied MOIs. Again, comfortably, increasing the MOI pushed VT signals toward higher fluorescence, with NTs decreasing proportionally (Fig. 2F). We posited that such increased “VT” signal could result from multiple phages adsorbing per cell. Indeed, microscopy visualization of ~500 single cells per treatment revealed that the number of detectable phages per infected cell increased proportionally to the MOI (Fig. 2F, G and  Supplementary Figs. S4–6). For example, of the tagged cells, few (~14%) cells exhibited multiple phages adsorbed at an MOI = 1, whereas those numbers increased drastically at MOIs = 2 and 4, where most (~55% and 67%) tagged cells exhibited multiple adsorbed phages per cell. As a negative control, we examined VT signals for a nonspecific phage, and this revealed that virtually all of the 545 single cells that were examined were nontagged (99.3%) even at an MOI = 10 (Supplementary Fig. S7). Presumably, the remaining ~0.7% of cells that appeared to have a phage adsorbed represent promiscuous, reversible binding to nonhost cells as is known to occur in other phage model systems [39]. Mechanistically, multiple phages can bind to a single host cell. For example, under very high-titer infection conditions (e.g., MOI = 100) phages can distribute over an entire cell surface [55], presumably accessing broadly distributed receptors [56]. Prior VT work has demonstrated strong VT signals under very high MOI (e.g., MOI = 1000) conditions [43], though no optimization experiments were presented to understand these patterns and the false positives that would result from free phages coincidently sorted (see further discussion later).

Finally, we re-evaluated the impact of cell physiology (e.g., early, middle, and late log phase host growth) and adsorption time (e.g., 20 min intervals from 0 to 120 min) on *Pseudoalteromonas* VT signals—and did so at two MOIs = 1 and 4, respectively (Fig. 2H). At both MOIs tested, growth phase was seen to impact the VT signals, with late log phase cells showing the highest fluorescent shift for VT cells in contrast to signals that were reduced in early log phase cells and nearly absent from stationary phase cells (Fig. 2H). This finding is consistent with our prior optimizations with *Pseudoalteromonas* phage-host model systems [39]. However, we observed that VT signals were optimal at 20 min after adsorption (see Methods) and, rather than stay high as we had previously observed, these experiments revealed that the VT signals were reduced by nearly half at subsequent time points. Though conflicting with our prior work [39], these current experiments employ hierarchical gating (Supplementary Fig. S1; see Methods), which we feel more appropriately quantify these patterns. This is because we interpret the signal reduction to be due to the lysis of first-adsorbed tagged cells and/or the injection of fluorescent DNA of the adsorbed virus(es) into cells as the latent period of phage HM1 for H71 cells under these conditions dictates [24]. Indeed, it has been reported that for phage lambda—*E.coli* system, the injection of fluorescent phage DNA followed by signal diffusion inside the cells decreased ~40% of the overall signal intensities of individual virus–host pairs [57].

Together, though an extensive set of experiments, these findings are largely confirmatory with our prior work characterizing *Pseudoalteromonas* phages [39]. However, and critically, our prior work failed to rigorously investigate these phenomena with respect to their (i) flow cytogram population signatures, (ii) single-cell microscopy imaging, and (iii) hierarchically gated tagged-cell timing estimates. We hope that these additional clarifications here provide a better mechanistic understanding of VT signals, and encourage wider adoption of this promising high-throughput method to identify viruses that infect a particular host.

### Introducing VT and grow: VT coupled to plate-based cultivation assays

Given this improved understanding of the VT signal, we next sought to expand VT to include an “and grow” capability to scalably capture and characterize viruses linked to hosts (conceptually presented in Fig. 1C). Pragmatically, this should also help resolve long-standing questions of (i) what fraction of VT cells lead to productive infections (i.e., does adsorption equal infection?, [45]), and (ii) whether sample processing (e.g., laser detection, sheath fluid growth inhibition [37, 58]) or cell density effects resulting from single-cell sorts [59, 60] would prohibit downstream growth assays.

To this end, we used the *Pseudoalteromonas*-virus HM1 model system to optimize sorting and growth conditions. Specifically, we wondered how many cells from sorted populations would be required to observe lysis (both dynamically, and terminally) under various MOI conditions. To test this, viral-tagged cells (the “VT” treatment) or nontagged cells (the “NT” treatment) were sorted into individual wells of a 96-well plate containing growth medium; fresh host cells were added, and growth-lysis curves were established by measuring optical density (OD) over time (see Methods). Treatment variables included the number of cells sorted (*n* = 1, 3, or 9) and infection conditions (MOI = 1 or 4), while controls included (i) NT cells to control for false-positive culture lyses by free viruses coincidently sorted with target cells, and (ii) sorting process controls against host cell lysis and growth in plates consisting of wells containing cultures with and without phage HM1, respectively. For all experiments, cells were infected during late-exponential phase for 10 min, followed by dilution to halt further infection, and centrifugation to remove free viruses (see Methods, [41]).

**Fig. 3:**
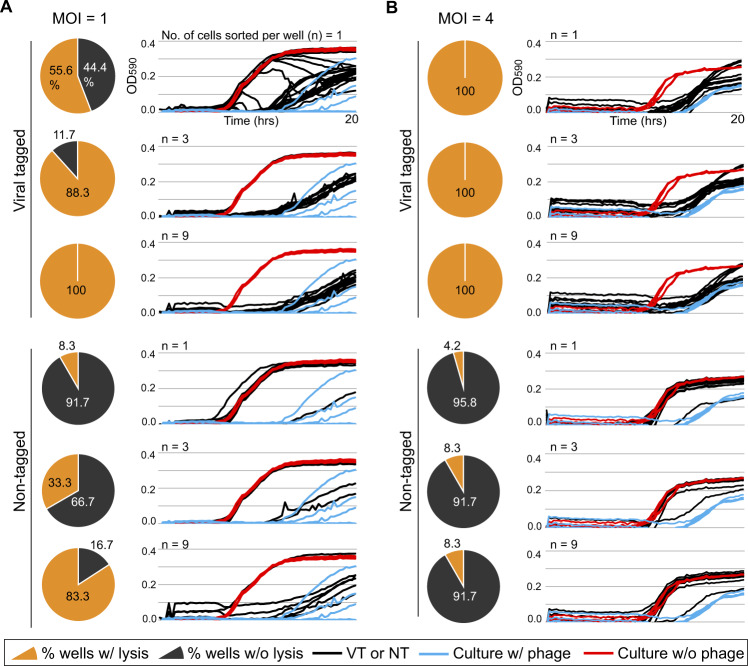
Evaluation of viral growth assay under various infection conditions. Two liquid cultures of *Pseudoalteromonas* strain H71 (10^5^/ml) in the late-logarithmic growth phase were infected by specific phage HM1 at MOIs of 1 and 4, respectively. From each infected culture, varying numbers of tagged (VT) and nontagged (NT) cells were sorted into individual wells of a 96-well plate containing growth medium followed by the addition of fresh host cells (10^4^ cells per well). Positive and negative controls (host culture with HM1 at an MOI of 0.1 and without HM1, respectively) were included in each plate (see Methods for details). From top to bottom, left to right in panels (**A**) MOI = 1 and (**B**) MOI = 4, respectively, pie charts depict the percentages of lysed (yellow) and nonlysed (gray) wells from the total wells containing the given numbers (*n* = 1, 3, and 9) of isolated VTs and NTs. Culture lysis for VT- and NT-containing wells was determined by comparing their growth curves (next to each pie chart, black lines) to those of negative (red) and positive controls (blue). The X-axis indicates the OD_590nm_ and the Y-axis, the time in hours.

We first analyzed the reduced-titer MOI = 1 infection. When only single cells were sorted, the growth curves from those wells as compared to those of phage-free controls, showed that more than half (56%; 20/36) of the VT wells with detectably reduced OD, whereas only a single NT well (8%; 1/12) showed such a decrease (Fig. 3A). This low rate of false-positive culture lysis in NT wells suggests that in most of the VT wells, progeny phages produced from an isolated parent VT—not free viruses―infect and lyse the host culture (For more details, see the burst size distribution of sorted single VTs below). Presumably, the 16 VT wells that did not lyse were due to one of the following: (i) reduced viability of isolated VTs through multiple steps of sample preparation or sorting with high sheath pressure [37, 58], (ii) possible reversible virus adsorption from the VT cell prior to well capture, and/or (iii) mis-diagnoses due to the weak fluorescent shift of singly-VT cells as is a known challenge in fluorescence-based cell sorting [58, 61].

To assess the MOI = 1 infections further, we evaluated the data for wells containing more than 1 cell sorted to each well. This revealed that sorting 3 or 9 cells improved the fraction of wells lysed in the VT treatments to 88 and 100%, respectively, but this came at the cost of increased false positives in the NT treatment (pie charts in Fig. 3A). The latter is likely due to the same challenges described above of differentiating the NT from VT populations when signal intensity was relatively low. Given the 96-well plate format, these experiments demonstrate the ability to follow growth kinetics for each well (time course OD figures in Fig. 3A). This revealed that single VT cell sorts had delayed lysis relative to the multiple-cell sorts and hints at the power such kinetics data could provide for scalably characterizing new *en masse* captured phage isolates from field samples. Stepping back, however, it is promising that the number of sorted cells per well, for both VT and NT wells, was linearly proportional to the percentages of lysed wells (*r*^*2*^ = 0.73 and 0.99), respectively (Supplementary Fig. S8). This suggests a robustness and repeatability for these experiments.

Beyond the fraction of the VT and NT wells displaying clear lysis, the kinetics of lysis—particularly for single-cell sorts—can be a valuable first read-out for variability in virus infection dynamics. To assess this in our dataset, we examined the kinetics of OD readings through 20 h (growth-lysis curves in Fig. 3A). Focusing on the 36 wells containing a single VT cell, 20 lysed (reported above), but their lysis kinetics drastically differed—some wells showed stepwise decreases after early increases in OD and the others a very low or no increase followed by the curve recovery. Similar lysis patterns have been observed in other phage-host systems, where host culture growth depended on phage concentration, with suppression of host cells increasing with higher phage titers and vice versa [62, 63]. Our observation of the well-to-well variation in culture lysis is likely due to different progeny production from isolated VT per well, relating to the stochasticity of viral infection [37, 64–67]. However, the stochastic infection alone cannot explain such diverse lysis patterns, given the random nature of diffusion and contact of progeny particles from infected cells to neighboring susceptible cells in the fluid (i.e., the host culture) [68, 69]. Either biological or physical infection process, or both, could impact varied lysis pattern. Further experiments are required to test this hypothesis (e.g., single-cell burst size assay, [37]; see below).

Finally, given that flow cytometric population separation was critical for optimizing lysis success and that simply sorting more cells comes at the cost of increased false-positive lysis, we next explored the impact of increasing the per-cell fluorescent VT signal with MOI = 4 infections. Indeed, sorting from these better-resolved populations improved our per-well lysis results as all of the VT wells lysed, and this was the case whether sorting 1, 3, or 9 cells per well (pie charts in Fig. 3B). For the NT wells, false positives were less problematic, but they did remain a minor problem as some wells (4–8%) lysed, and this increased in the multiple-cell sorted wells. Though VT and NT populations are likely better resolved, thereby reducing false-positive lysis in the NT wells from the MOI = 1 infections, presumably the higher MOI infections lead to free viruses being coincidently co-sorted in the sort droplets. Notably, the kinetic read-outs (growth-lysis curves in Fig. 3B) were relatively invariable, possibly suggesting that the much higher number of viruses-per-cell in these infections obscured virus-to-virus variability in life history traits [66, 67, 70].

Together, these experiments provide strong baseline data for assessing the impact of VT signal quality, MOIs, and growth data and hint that the approach may also open up new windows into variation in trait space across virus isolates.

### New biology enabled by viral tag and grow: a window into “viral individuality”?

A major challenge in viral ecology is scaling from the handful of viruses that might be well characterized to the millions of virus types in an average seawater or field sample. While diversity surveys have come a long way (e.g., hundreds of thousands of viruses in a single study [23]), the pragmatic challenges of taking physiological measurements across many viral isolates leaves modeling efforts with very little empirical data on virus life history traits, severely bottlenecking the viruses brought into predictive models [71]. Further, microbiologists have revealed that even among “clonal” isolates, there can be remarkable phenotypic heterogeneity, or “microbial individuality” [72–74]; does the same exist for viruses? Hints that there is such “virus individuality” among DNA viruses, including phages, are emerging with data demonstrating variability in single-cell burst size (progeny per infected cell), with up to ~100-fold differences and these differences attributed to stochastic events such as variation in starting points in cell size, growth stage, and resources [37, 64–66].

Of particular interest in understanding ‘virus individuality’ are recent single-cell analyses developed for a *Synechococcus* phage-host model system that revealed a wide range of burst sizes (from 2 to 200 infective viruses/cell) within a laboratory clonal isolate [37]. Methodologically, this approach sorts cells—infected or not—into wells (e.g., of a 96-well plate) and follows their infection dynamics. This has the benefit of assessing a single cell’s growth-lysis curve in each well. However, a drawback is that experiments are more conveniently done at high MOI conditions (e.g., an MOI = 3 was used) to get larger numbers of wells lysing among the randomly sorted cells (see Methods). Increasing MOI will lead to more virus-containing and, therefore, lysing wells, subsequently greatly increasing the number of cells with multiple viruses attached such that it will confound measurements of lysis dynamics since they will be a function of both virus-to-virus ‘individuality’ and an unknown, but variable per-cell MOI [70, 75].

**Fig. 4:**
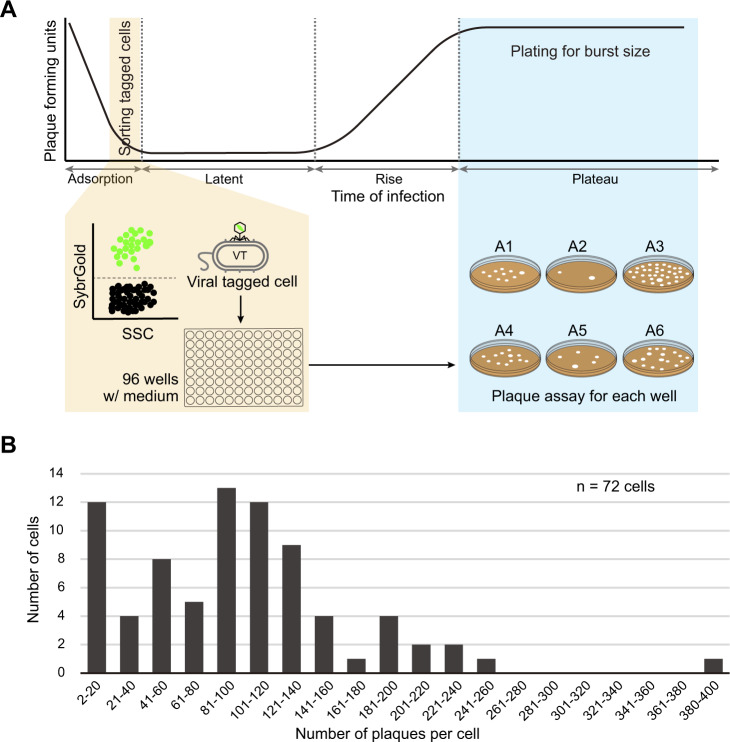
Distribution of virus burst sizes per single viral-tagged cell. **A** Schematic overview of single-cell assay for viral burst size determination by viral tagging and grow. In the latent period of infection, single viral-tagged cells (VTs) were sorted by flow cytometer from *Pseudoalteromonas* sp. H71 cells infected by phage HM1 at an MOI of 0.5 (see Methods for details). Following sorting single VTs into different wells of the 96-well plate containing growth medium (MSM), the plate was incubated to allow for viral progenies to release from infected cells. The number of viruses produced per VT was then determined by the number of plaques per poured plate using the traditional plaque assay. **B** Distribution of viral burst size from individual tagged cells. The number of progeny viruses (X-axis) per cell (Y-axis) are represented in bins of 20, with the exception of the first bin excluding single plaques. The number (*n*) of individual tagged cells assessed is represented at the top right corner.

Inspired by this latter work, we sought to improve such single-cell growth-lysis assays in ways that might leverage the scalability of VT + Grow. For these experiments, we wanted to reduce the MOI (to MOI = 0.5) since theory predicts that most (77%) of the infected cells would be singly infected (Poisson distribution), but keep it high enough to have a reasonably separated VT cell population (see Methods). After cells and viruses were mixed, individual VT cells were sorted into different wells containing growth medium, plates were incubated to allow lysis of the single sorted VT cell, and the number of plaques per well were determined by pour plate plaque assays (Fig. 4A; see Methods for details). This operationally single-cell burst size assay showed a wide range of infective viruses per cell (2 to 397, X-axis) from a total of 72 individual cells assessed (Y-axis) (on average = 100; Fig. 4B), with similar average population burst sizes of 110 ± 15 [24]. Though a clonal virus isolate, these findings suggest, just as seen for cyanophages [37], that stochastic events must dictate the specific burst size for any given interaction. However, unlike the prior work, it is unlikely that cells with multiple viruses adsorbed any of this signal since such events should be much rarer at an MOI = 0.5 instead of MOI = 3. This suggests that these stochastic events are of a biological nature, which we posit might mechanistically result from the timing of initial virus–host interactions and/or cell-to-cell or virus-to-virus variation in nonheritable traits such as per-cell nutrient stores. If we interpret such infected cell variability as ecologically relevant variation in “virocells” (*sensu* [13, 76, 77]), then these findings open a window into “virus individuality” via a more scalable and controllable characterization approach than previously available.

### Limitations and future development opportunities for VT and Grow

Though these efforts provide a more robust foundation for broadening the use of VT related methods, there remain challenges. *First*, researchers must be aware that VT is not a simple method, and its success depends on instrument calibration and ultraclean sample processing to establish maximally separated VT and NT populations (see the link below for details on flow cytometric setup and optimization). *Second*, sorting purity, particularly in field applications, will be challenged by suboptimal VT flow cytometric signatures, e.g., mis-identification of NT cells. Though this can be overcome with very high MOI infections (e.g., 1000 viruses per cell, [43]), two issues remain: (i) the effective MOIs cannot be measured in field samples (and thus, unknown), and (ii) at such high MOIs, the experiments will suffer from coincident sorting of free viruses that will increase false positives. Another factor that could affect sorting purity is nonviral DNA in the environmental sample, whether it is associated with bacterial cells or not, which could be coincidently sorted. It is thus necessary to ensure that prior to any VT work, environmental samples are properly processed or treated for the removal of nonviral genes and other materials (e.g., filtration and/or centrifugation). Fortunately, the “and grow” approach added to VT provides an additional screening step whereby false-negatives and false positives can be discerned via growth-lysis monitoring. Further, the “and grow” component, a plate-based assay, enables faster and more scalable lysis screening (e.g., 96-well format) than the time- and labor-intensive traditional plaque assay [62, 63]. *Third*, viral aggregates that alter the effective MOI infection conditions could lead to confounding results when comparing results across laboratories. Here, we invite efforts to find and optimize approaches to reduce viral aggregates (e.g., detergents, sonication, syringe pumping), and until viral aggregates are eliminated, to microscopically examine the state of free viruses in new sample types, particularly for outlier results. *Fourth*, the methods remain dependent upon a cultivable host, and though VT has been applied to multiple heterotroph and cyanobacterial phage-host pairs [39], two big unknowns remain: (i) how will the “and grow” processing impact growth of these strains, and (ii) will non-marine model systems be amenable to these approaches. The in-depth optimizations presented here for a *Pseudoalteromonas* phage-host model system serve a foundation for understanding other target virus–host pairs. To this end, we suggest deep investigation for any new model systems being studied, and as information becomes more broadly available, invite a community-standards and benchmarking approach to determine ideal setups for infectious conditions (e.g., growth curve, MOIs) and instrumental parameters. To facilitate this, we have established a VT forum on the Viral Ecology VERVE Net living protocols at protocols.io (below) as a way to empower and broadly engage researchers interested in these new methods and the many variants that could blossom from this base. Specifically, the details for viral and bacterial sample processing can be found at https://www.protocols.io/view/viral-tagging-and-grow-a-scalable-approach-to-capt-bwutpewn?form=MY01SV&OCID=MY01SV and for flow cytometric optimization at https://www.protocols.io/view/bd-influx-cell-sorter-start-up-and-shut-427down-for-v-bv8cn9sw. Both protocols provide additional notes for critical steps to improve methodological reproducibility and/or sensitivity, and particularly for the latter, it will be updated regularly to better optimize, calibrate, and standardize a flow cytometer.

## Conclusions

Here we take an important step forward in the quest to experimentally link viruses to their hosts by improving understanding of VT, and establishing a new variant (VT + Grow) that offers scalable virus capture and characterization capabilities for cultivated host cells. This advance comes at a time where significant and diverse efforts are improving experimental measurements and scalability of virus–host linkages—e.g., VT [38, 39, 43, 48], epicPCR [40], AdsorpSeq [42], and microfluidic digital PCR [78]. However, this VT + Grow approach provides the added benefit of “capture” whereby the method, though cultivation dependent, can lead to rapid stocks of clonal virus isolates that are demonstrably capable of infecting a cultured “bait host” of choice. Further, under appropriate sorting conditions, scalable growth kinetic measurements are captured as well—either directly, or, for more certainty, coupled to traditional plaque assays on the back-end after the scalable front-end virocell capture. In all, VT + Grow offers a new tool in the toolkit that we hope will inspire community-driven innovations and applications that are needed to better “see” the power of viruses as hidden movers and shakers in our microbiome-impacted world.

## Materials and methods

### Growth and maintenance of bacteria and bacteriophages

*Pseudoalteromonas* phages and their hosts used in this study were recovered from a Helgoland collection in 1990, Germany [79, 80] and reestablished clonally, as previously described [24, 39, 81, 82]. For details on the culture conditions used, see the Supplementary Information.

### Viral staining and washing

Our viral staining and washing procedure was modified from a previously published VT approach [38, 39]. Details are represented in the protocols.io (https://www.protocols.io/view/viral-tagging-and-grow-a-scalable-approach-to-capt-bwutpewn?form=MY01SV&OCID=MY01SV)

### Microscopic characterization of virus–host pairs

We visualized virus–host pairs by microscopy at the single-cell level. This experiment aimed to check whether the *Pseudoalteromonas* virus-positive signals in VT would correspond to the adsorption of viruses to hosts. To test this, we infected *Pseudoalteromonas* sp. H71 separately with host-specific myovirus HM1, and nonspecific siphovirus HS8. Through quantification of the relative fractions of virus-tagged (VTs) and nontagged cells (NTs) in each virus-bacterium mixture at various MOIs, we compared them to the percentages of green-shifted and non-shifted populations of VT, respectively (see below). Samples for both microscopic and VT inspections were processed in parallel at both the same MOIs to ensure similar contact rates [37].

Phages HM1 and HS8 were stained and washed identically (above). *Pseudoalteromonas* sp. H71 cells were counterstained with 4′,6-diamidino-2-phenylindole (DAPI, 10 µg ml^−1^ final concentration, Sigma-Aldrich, Cat. No. D5942, an excitation peak at 359 nm and an emission peak at 457 nm) for 20 min in the dark. Stained cells were washed three times by centrifugation at 8000 × *g* for 5 min at 4 °C and resuspended in PZM (*Pseudoalteromonas*-Zobell Media, containing approximately half the concentration of nutrients [24]) to remove excess dye molecules. Bacterial cells were then mixed separately with fluorescently-labeled viruses HM1 and HS8 to final MOIs = 1, 2, and 4 and 2, 5, and 10, respectively (six samples in total). After incubation for 10 min followed by three repetitions of centrifugation (16,000 × *g* for 1 min at room temperature) and resuspension in MSM (450 mM NaCl, 50 mM MgSO_4_ · 7H_2_O, 50 mM Tris-HCl, pH 7.5), each sample was immediately fixed with glutaraldehyde (0.25% final concentration) and ascorbic acid antifade solution (1% final concentration, [83]). In parallel, to compare the removal of free viruses, we additionally prepared the sample (phage HS8 and H71 cells) without centrifugation and resuspension (Fig. S7). A volume of 2 µl from each sample was smeared on Poly-L-lysine glass slide (Thermo Scientific, Cat. No. J2800AMNZ) and incubated in the dark for 30–60 min for immobilization of cells. Samples were observed under an automated epifluorescence microscope (Zeiss Axioplan2 imaging, Carl Zeiss, Oberkochen, Germany) equipped with a monochrome camera (AxioCam mRm, Carl Zeiss Microimaging GmbH, Göttingen, Germany). No movements of both cells and viruses were apparent. Microscopy signals were interpreted as follows: blue signals without green were interpreted as nontagged cells (NTs), spot-like green signals on the margins of blue signals were interpreted as virus-tagged cells and spot-like green signals outside cells were interpreted as “free” viruses. A total of ~500 cells were counted per sample to quantify the percentage of VTs and NTs, respectively. For VTs, based on the number of detected viral signals, we further categorized them into multiple (≥2) or single-virus-tagged cells.

### Optimization of flow cytometer for cell sorting

Bacterial and viral samples were examined using a BD influx cell sorter (Becton Dickinson, San Jose, CA). The instrument is equipped with two high-power lasers at 488 nm (blue) and 642 nm (red) and nine optical detectors to analyze the size, granularity, and seven fluorescences per cell. Each detector uses a high-performance photomultiplier (PMT) that can amplify low-intensity signals of the nano-sized particles such as viruses [50, 84]. Additionally, this influx sorter has the capability of 2- to 6-way population sorting into tubes and single-cell sorting directly into plates or slides. The fluidic system was only run using sterile solutions as a sheath (i.e., 0.02-µm-filtered and autoclaved MSM) and is always cleaned and air dried before shutdown. Details on the instrument setup (e.g., start-up, laser/stream alignment, drop delay determination, dry/wet shutdown, and maintenance) are presented in the protocols.io (https://www.protocols.io/view/bd-influx-cell-sorter-start-up-and-shut-down-for-v-bv8cn9sw).

To identify the cells, we used hierarchical gating. This sequential gating strategy can detect the subpopulations, daughter gate, from the cells of interest, parent gate [50]. A parent gate was drawn on *Pseudoalteromonas* sp. H71 cells in the forward (FSC) vs. side scatter (SSC), from which viral-tagged (VTs, green positive) and nontagged (NTs, green-negative) cells were further gated as a sub-fraction in the green fluorescence vs. SSC. Events were detected using a forward scatter trigger, and data was obtained in logarithmic mode then analyzed with BD FACS software version 1.2.0.142 (Becton Dickinson, San Jose, CA). The trigger and the PMT voltages for relevant parameters (FSC and SSC) were adjusted to avoid the overlap of the cell signals with background noises that could come from the instrument (electronic noise), micro-particles in the buffer, and/or cellular debris [50] (Fig. S1). Samples were typically run with ~10^5^ ml^−1^ cells. Green fluorescence was detected using a 542/27 band pass filter with an amplified PMT.

For single-cell sorting of VTs and NTs, we used the single sort mode of “1.0 drop purity”. Since a low flow rate of the sample provides sufficient separation between sorted particles, it can reduce the possibility of isolating multiple particles in a single droplet [50, 84]. Particularly for viruses and bacteria, the event rate of 40–50 (i.e., no. of particles detected per second) was successfully used for single-virus sorting [85] and less than 300 for bacteria [86]. Thus, to prevent the coincidence of free viruses sorted with VT or NT cells, which would be too close to be separated, we adjusted the event rate for sorted particles from the VT and NT cell populations to ~40 events per second. The estimated ratio of sorted particles to generated drops (piezoelectric at 43.2 kHz, above) was ~1/1180 which ensured a separation enough between sorted particles [85]. Flow cytometric sorting is challenging, particularly when fluorescence-positive populations are smaller than their negative counterparts, and their signals are poorly separated from each other [61]. Thus, to maximize the sorting purity of false-positive population, we used the biased gating strategy, as previously described [61]. Fine alignment of the 96-well plate was performed by visually inspecting the deposition of droplets into the center of each well. Prior to sorting, 100 µL of Zobell media was added to each well to prevent desiccation of sorted droplets during processing. Using the “sorting limit” to control the number of events sorted per well, each row was seeded with one, three, or nine cells per well from VT and NT populations. After sorting, each of these 84 wells (12 × 7) was inoculated with approximately 10^4^ *Pseudoalteromonas* sp. H71 cells in Zobell medium. The remaining row H contained four wells of a media blank (200 µl PZM buffer only), four wells of a negative control (10^4^ H71 cells without HM1), and four wells of a positive control (10^4^ H71 with HM1 at an MOI = 0.1).

### Viral tagging and grow

VT experiments were performed as previously described [38, 39]. Briefly, stained and washed phages HM1 and HS8 (above) were separately mixed with 10^6^ *Pseudoalteromona*s sp. H71 in exponential phase at varying MOIs = 1, 2, and 4 and 2, 5, and 10, respectively. Small (<200 µl) infection volumes were used to maximize the contact rate of viruses and cells while reducing the amount of biomass needed to conduct each experiment. After 10 min incubation of phages with H71 cells, MSM buffer was added to bring the infection volume up to 1 ml, and the virus–host mixtures were immediately centrifuged (12,000 × *g* for 1 min at room temperature) and resuspended in sterile MSM medium to remove free viruses; this process was repeated thrice. To decrease coincident sorting of multiple cells per droplet and to achieve optimal spacing between sorted cells, the infected cells were diluted to final concentrations ranging from 5 × 10^5^ ml^−1^ to 10^6^ ml^−1^. As a negative control, the MSM medium without viruses was prepared identically to the stained and washed virus sample, which controlled for free dye creating the false-positive green-positive populations [38, 39]. Following FCM sorting of cells into the 96-well plate (see above), bacterial growth was continuously monitored by measuring OD_550_ nm at 15-min intervals for 24 h. Growth curves were obtained by plotting OD following baseline adjustment of the blank against time. We then compared the lysis patterns of the wells containing sorted VT and NT cells with a varying number to those of positive and negative controls (above). All assays were performed with three replicates. Details can be found in the protocols.io (https://www.protocols.io/view/viral-tagging-and-grow-a-scalable-approach-to-capt-bwutpewn?form=MY01SV&OCID=MY01SV).

### The impact of cell physiology on viral tagging signals

We used VT to detect adsorption dynamics of phage HM1 to *Pseudoalteromonas* H71 under various infection conditions (e.g., multiplicity of infections, MOIs) and host growth culture (e.g., early log, middle log, and stationary phases) as previously described [39], with some modifications. Details are represented in the Supplementary Information.

### Per-cell variability in viral progeny production revealed by viral tagging and grow

Per-cell virus yield (burst size) is more prominent when host cells are infected by single phage particles, because multiple phages co-infecting a host can confound intracellular phage’s production through either competitive or cooperative interaction, or both [66, 70, 75]. Thus, it is critical to adjust the ratio of phages to host cells (e.g., the multiplicity of infection, MOI) to make single phages infect single cells [66]. However, for single-cell assays using the flow cytometer, low-MOI infections to obtain singly infected cells result in the plate that mainly contains uninfected cells (90.5% wells at an MOI = 0.1, Poisson distribution). At such low MOIs, it is challenging to obtain a reasonable number (*n* = 50–100) of infected cells for analysis [37, 87]. Due to this technical limitation, the previous flow cytometric study for cyanophages has used a high MOI = 3 [37] to increase the chance of more wells having infected cells.

VT and grow (VT + Grow) enables to selectively isolate virus-adsorbed cells from the infected culture (Fig. 1). This allowed us to use lower MOIs than 3 (above) for burst size assessment of phage HM1 per *Pseudoalteromonas* H71 cell. We tested two MOIs of 0.1 and 0.5, both of which are expected to contain <40% infected cells, but >75% of them are singly infected (Poisson distribution). Of these, we chose an MOI = 0.5 due to almost no signal shift (1–2%) of the viral-tagged population at an MOI = 0.1 (data not shown).

Burst size estimation by VT + Grow consists of two steps: (i) sorting of individual cells into the medium-containing wells and (ii) plating of viral progeny produced from each well (Fig. 4A). To determine the burst size by VT + Grow, we setup the timing for sorting and plating based on the prior knowledge of the one-step growth curve of phage HM1 for H71 cells [24], as previously described [37]. Briefly, phages HM1 were fluorescently stained and washed (see Viral staining and washing) and then, incubated with H71 cells (see VT and grow) at an MOI = 0.5 (above). From the virus–host mixtures, viral-tagged, and nontagged populations were detected through the hierarchical gating, from which individual tagged cells were sorted into different wells of the 96-well plate containing PZM (see Optimization of flow cytometer). For phage HM1 infection of H71 cells, the initial plateau of virus release occurred by 55–75 min after virus addition [24]. Therefore, the 96-well plate containing sorted tagged cells was incubated for 40–50 min, for a total process duration of 65–75 min after virus addition (10 min for virus incubation with host, 5 min for centrifugation and resuspension, and 10 min for flow cytometric detection and sorting). After that, the content (200 µl) of each well was immediately mixed with 400 µl of liquid bacterial culture (~10^8^ cells/ml) and plated using the agar overlay assay by adding 3.5 ml of molten soft agar (0.5% agar in PZM, 50 °C), as previously described [82]. The MSM medium with and without phage HM1 were prepared as positive and negative controls, respectively. After overnight incubation, the number of plaques for each plate was determined using an inverted microscope.

## Supplementary information

Supplementary Information
