# Supplementary material for: Viral tag and grow: a scalable approach to capture and characterize infectious virus–host pairs
Source: ISME Commun. 2022 Feb 1;2:12. doi: 10.1038/s43705-022-00093-9 (PMC9723727; doi:10.1038/s43705-022-00093-9)
Supplement: Supplementary file 1 — Supplementary Information [file 43705_2022_93_MOESM1_ESM.pdf]

Supplementary Information for

Viral tag and grow: a scalable approach to capture and characterize infectious virus-host pairs

Ho Bin Jang[1], Lauren Chittick[1], Yueh-Fen Li[1], Olivier Zablocki[1], Courtney M. Sanderson[1], Alfonso Carrillo[1], Ger van den Engh[2] & Matthew B. Sullivan[1, 3, 4]#
[1]Department of Microbiology, The Ohio State University, Columbus, Ohio, 43210, USA
[2]Marine Cytometry Inc, Concrete, Washington, 45770, USA
[3]Department of Civil, Environmental and Geodetic Engineering, The Ohio State University, Columbus, Ohio, 43210, USA
[4]Center of Microbiome Science, The Ohio State University, Columbus, Ohio, 43210, USA

#Address correspondence to Matthew B. Sullivan, sullivan.948@osu.edu, +1-614-247-1616

Supplementary Text

Materials and Methods

Growth and maintenance of bacteria and bacteriophages

Pseudoalteromonas sp. strain H71 were stored with 50% (v/v) glycerol in liquid nitrogen, and streaked out on Zobell media plates. Individual colonies were inoculated in PZM media (*Pseudoalteromonas*-Zobell Media, containing approximately half the concentration of nutrient, (1)) and incubated overnight at 21℃, shaking at 150 rpm, the day prior to experiments. In the hours preceding infection for either viral tagging or epifluorescent analysis the overnight culture was then transferred 1:100 into fresh PZM and incubated until it reached exponential phase.

Phage HM1 was chosen for protocol optimization dye to its high infective titer (2×10^{10} pfu ml^{-1}) on H71. Lysates were kept at 4℃ in sterile 0.1 µm-filtered MSM buffer (450 mM NaCl, 50 mM $MgSO_4 \cdot 7H_2O$, 50 mM Tris-HCl, pH 7.5) and propagated and recovered using the soft agar overlay technique (2). Following resuspension in sterile buffer, this viral lysate was then quantified by plaque assay for infective particles, and SYBR wet mount enumeration for total viral particles (3). *Pseudoalteromonas* phage HS8 and its host strain 13-15 were prepared in the same way for phage HM1 and H71 cells (above).

Viral adsorption kinetics experiment

Because sorting virus-tagged cells is contingent on the adsorption of fluorescently-stained virus particles, timing of the phage infection is critical. To establish a time window for sufficient adsorption of phages well before cell lysis (4), we performed a traditional adsorption assay (1, 3). In three biological replicates, a transfer culture of *Pseudoalteromonas* sp. H71 in the mid-exponential phase ($\sim 5 \times 10^{7}$ ml^{-1}) was infected with phage HM1 at a multiplicity of infection (MOI) = 1. Total phage and cell titer were sampled immediately after infection to confirm the MOI, and subsequent free phage and cell samples were collected every 5 min for 40 min. Free phage samples were diluted 100-fold and 0.2 µm filtered to remove bacteria; samples were diluted in artificial seawater (psu of 26) for plaque assays to determine the number of free phages as plaque-forming units (PFUs) ml^{-1} suspension. The free phages percentage was calculated from the decline in free phages at each time point relative to the initial input number of phages at time zero.

The impact of cell physiology on viral tagging signals

The stained and washed phages HM1 (see Viral staining and washing above) were mixed with bacteria H71 ($\sim 10^{6}$ cells) in the early log, late log, and stationary phase at MOIs = 1 and 4, respectively. For each sample, with the hierarchical gating (details can be found in the protocols.io, https://www.protocols.io/view/viral-tagging-and-grow-a-scalable-approach-to-capt-bwutpewn?form=MY01SV&OCID=MY01SV), the percentages of tagged populations were measured at the time point (every 20 minutes up to 120 minutes) after incubation for 10 min followed by three repetitions of centrifugation and resuspension in sterile MSM medium to remove free viruses. Each test was done in duplicate. Sample line was cleaned for 1 minute by backflush between samples.

Supplementary Figures

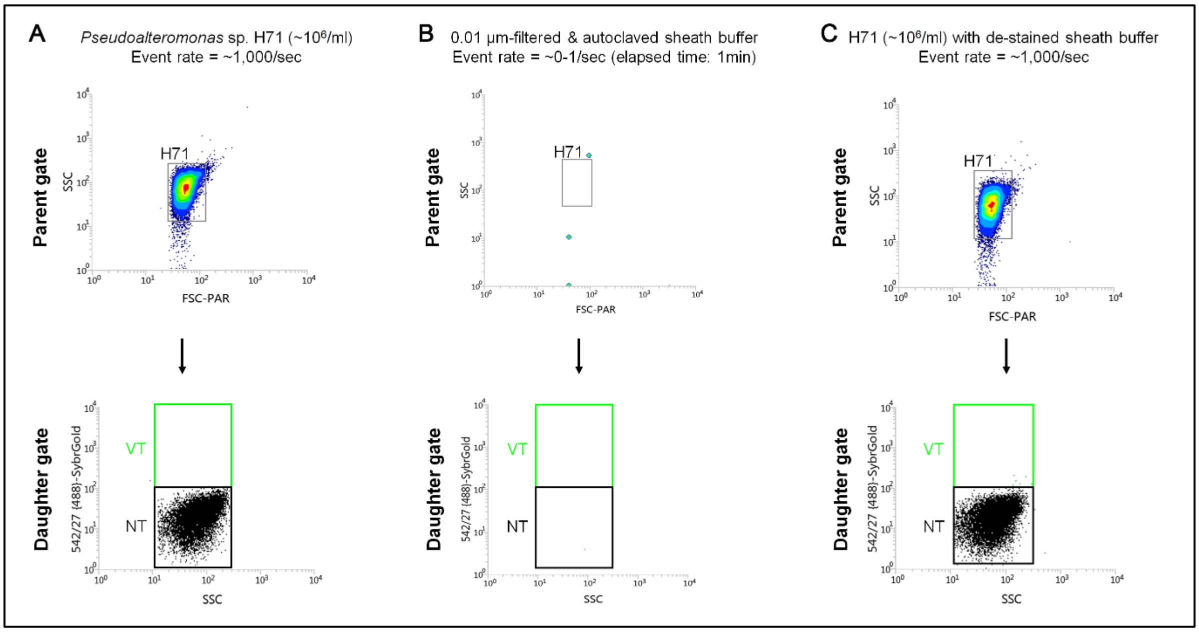

Fig S1. Flow cytometric optimization of viral tagging signal detection. A *Pseudoalteromonas* strain H71 cells (~10^6/ml) were detected with a tight gate (event rate: ~1,000 events/second) using forward (FSC) versus side scatters (SSC) (Upper). From this parent gate, the virus-tagged (VTs) and nontagged populations (NTs) were sub-gated, using green fluorescence (542/27 nm, SybrGold) and SSC (Lower). **B** Autoclaved and filtered sheath buffer with H71 cells on the influx. Note that there is the non-overlap of cell populations (gated) with background noises (electronic noise and/or microparticles in the sheath buffer) in a parent and two daughter gates (VT and NT populations, respectively). Detected events were shown for 1 min of sample running (elapsed time) with an event rate of 0 to 1/sec. **C** H71 cells with stained and destained sheath buffer (see Methods for details) on the influx. This controlled for possible false-positive events that could result from free dye-based SYBR staining, which in these experiments was negligible (~0.02-0.1% of the population).

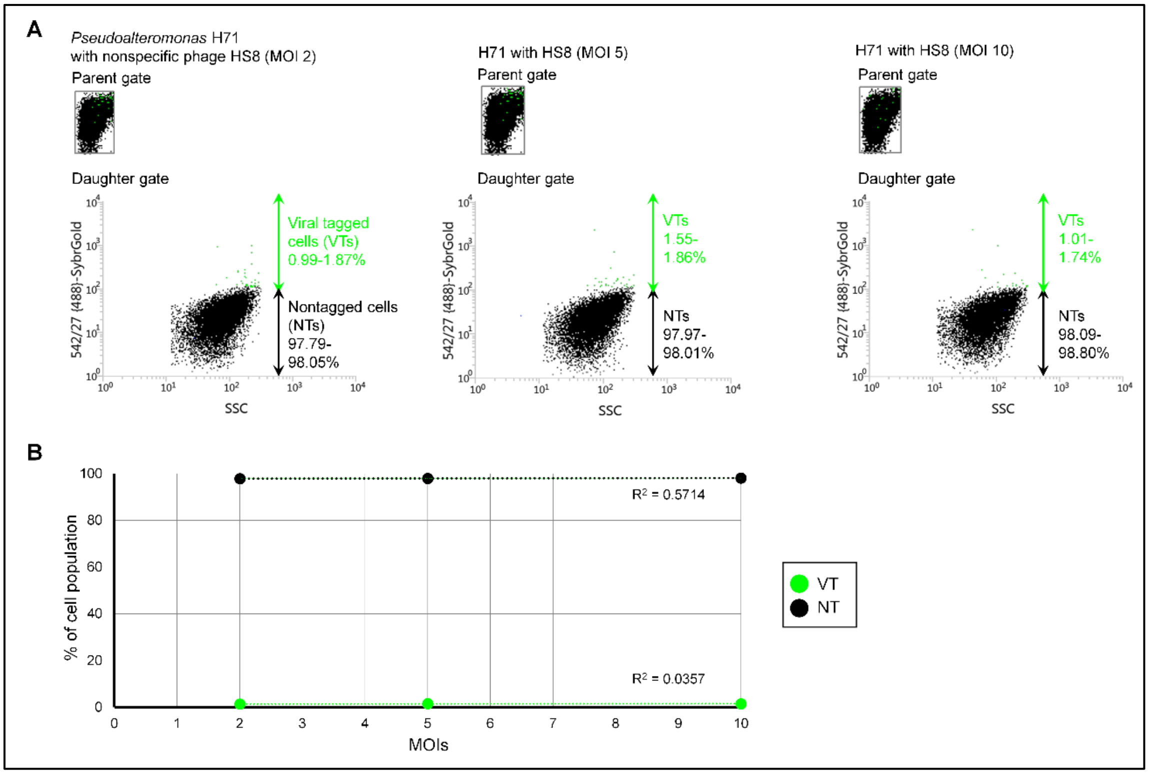

Fig S2. Viral tagging signals for infection of nonspecific phage HS8 to *Pseudoalteromonas* strain H71 cells. H71 cells were first gated using forward (FSC) versus side scatters (SSC) (see Fig S1 for hierarchical gating strategy). From this parent gate for H71 cells (upper in each panel), green-positive (viral tagged, VT) and green-negative (nontagged, NT) populations are sub-gated using green fluorescence (542/27 nm, SybrGold) and side scatter (SSC). **A** the percentages of VT and NT populations (lower right in each panel) and their corresponding plots (left) from H71 cells infected by nonspecific phage HS8 at MOIs of 2, 5, and 10 (from left to right in each panel), respectively. For viral tagging signals from specific phage HM1 and H71 cells at various MOIs, see Fig. 2d. **B** linear regression analysis to investigate the relationship between MOIs and the percentages of VT and NT populations for nonspecific phage HS8 at MOIs of 2, 5, and 10 (from left to right), respectively. X-axis indicates the MOI and Y-axis, the percentage of VT or NT population. R-square value is represented in each analysis.

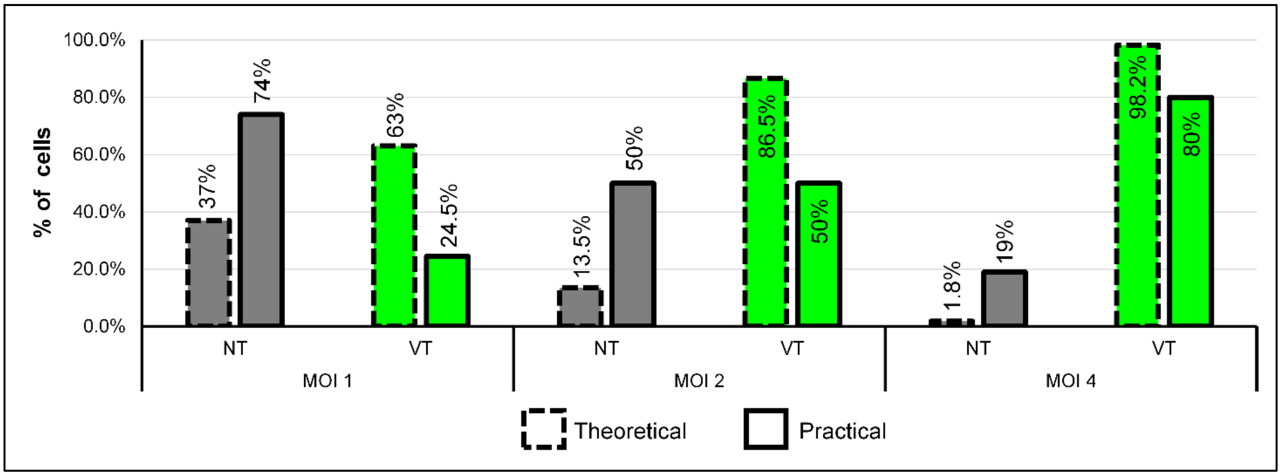

Fig S3. Theoretical vs. practically observed fraction of tagged cells across MOI-varied experiments. Comparisons of the percentages of viral tagged (VTs) and nontagged cells (NTs) obtained by theoretical calculation (Poisson distribution) and practical (flow cytometry) observations at MOIs of 1, 2, and 4, respectively. Black and green bars represent the NTs and VTs at the respective MOIs. For practical infection (phage HM1 to *Pseudoalteromonas* sp. H71) observed by flow cytometry, the averaged percentages of VTs and NTs are shown.

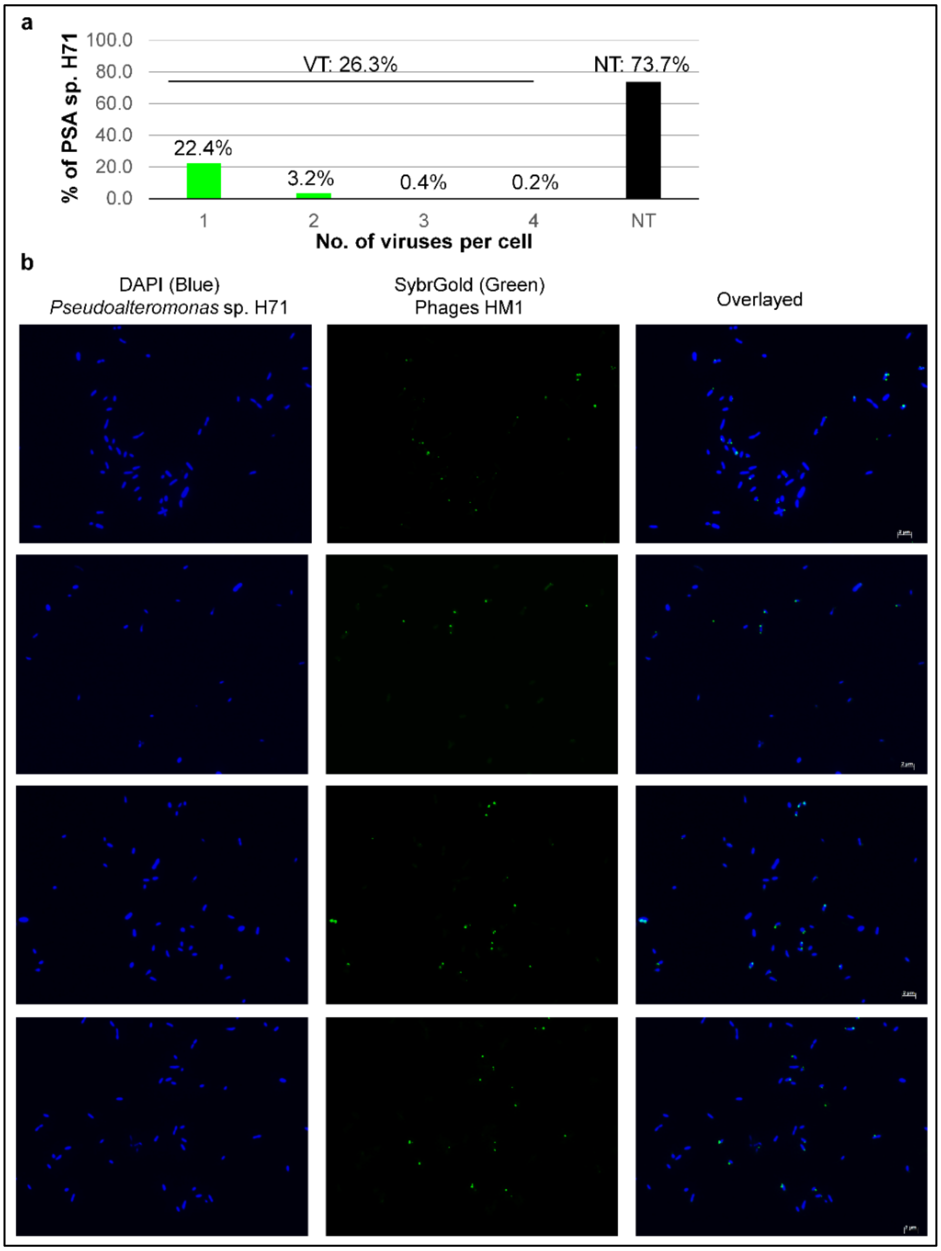

Fig S4. Microscopic evaluation of viral-tagging at MOI=1. A Microscopic quantification of percentage fractions of virus-tagged (VTs) and nontagged cells (NTs) from *Pseudoalteromonas* strain H71 infected by specific phage HM1 at an MOI of 1. From a total of 673 cells, virus-tagged cells (green-colored viral signals on the margin of blue-colored cell signals, see panel B and nontagged cells (blue signals without green signals, see panel b) were quantified, respectively. Numeric values (1, 2, 3, and 4+) in the X-axis correspond to the number of green-colored phage particles that were counted on the margin of DAPI-stained cells. **B** Representative microscopic images of *Pseudoalteromonas* stain H71 infected by phage HM1 at an MOI of 1 were shown due to space limitations. Left column: host cells stained with DAPI (blue), Middle: phage HM1 with SybrGold (green), Right: overlay of host cells and viruses. The scale bar indicates 2 µm.

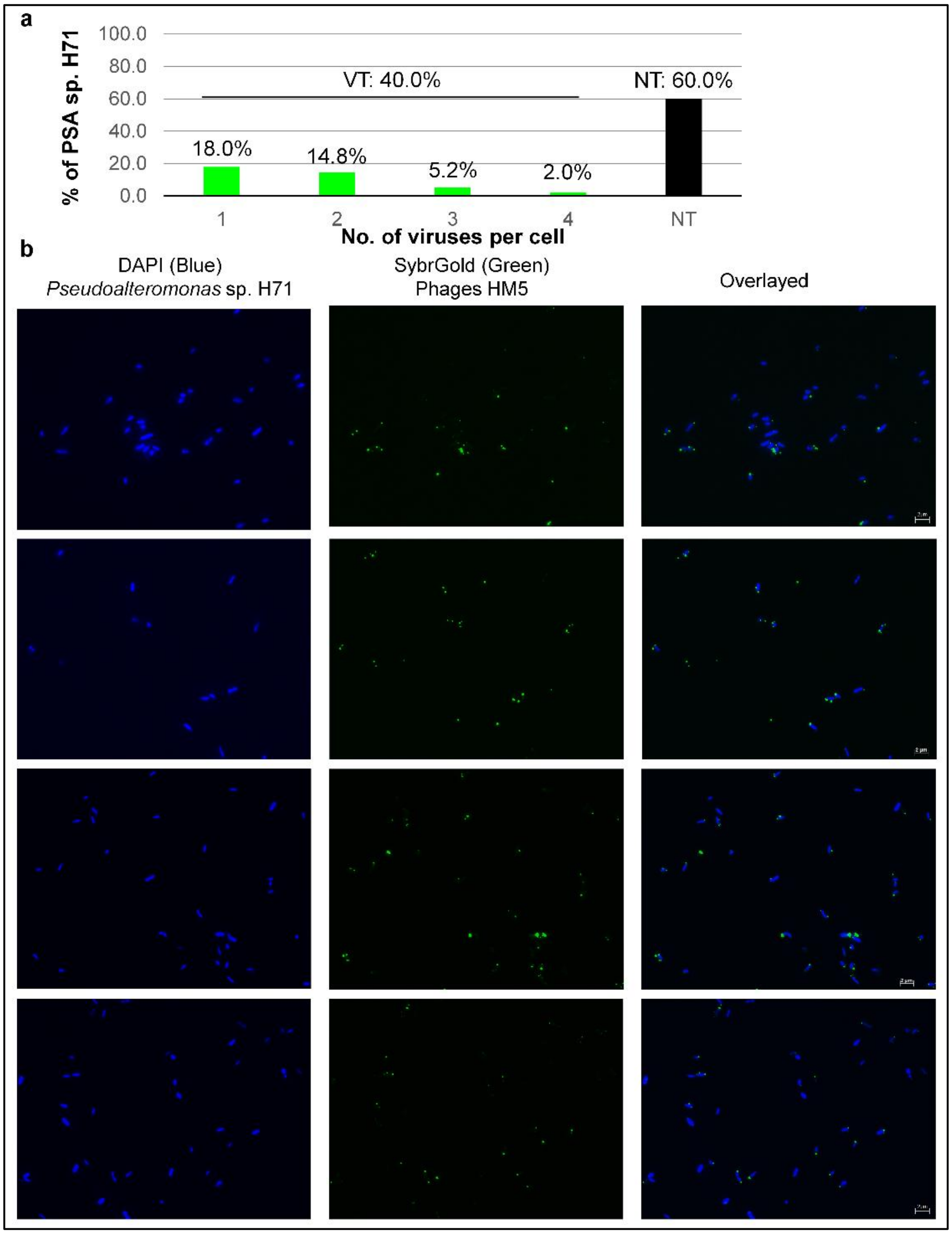

Fig S5. Microscopic evaluation of viral-tagging at MOI=2. A *Pseudoalteromonas* strain H71 infected by specific phage HM1 at an MOI of 2. From a total of 530 cells, the percentage fraction of virus-tagged (VTs) and nontagged cells (NTs) were quantified. **B** Representative microscopic images were shown due to space limitation. Figure details as in Fig S4.

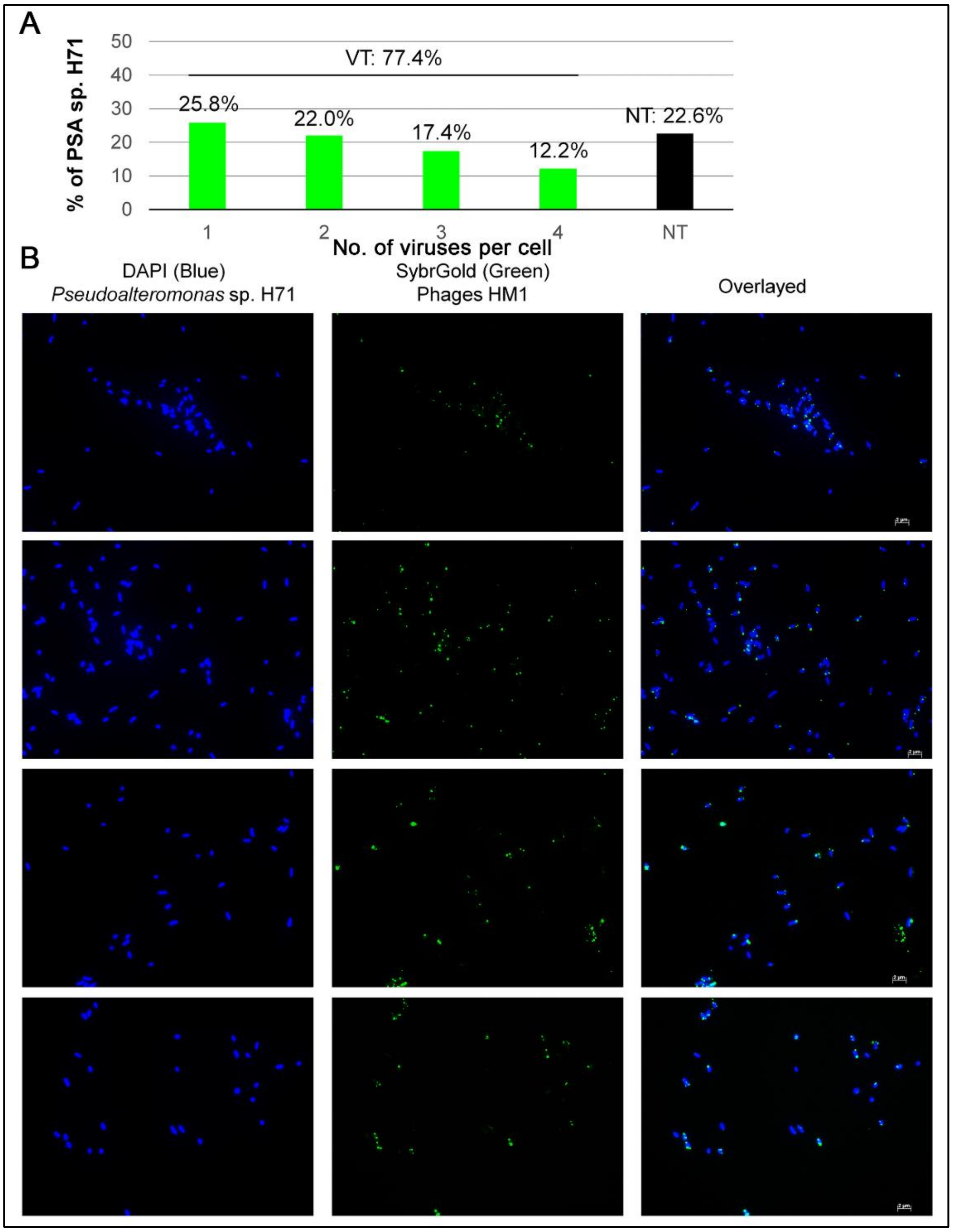

Fig S6. Microscopic evaluation of viral-tagging at MOI=4. A *Pseudoalteromonas* strain H71 infected by specific phage HM1 at an MOI of 4. From a total of 675 cells, the percentage fraction of virus-tagged (VTs) and nontagged cells (NTs) were quantified. **B** Representative microscopic images were shown due to space limitation. Figure details as in Fig S4.

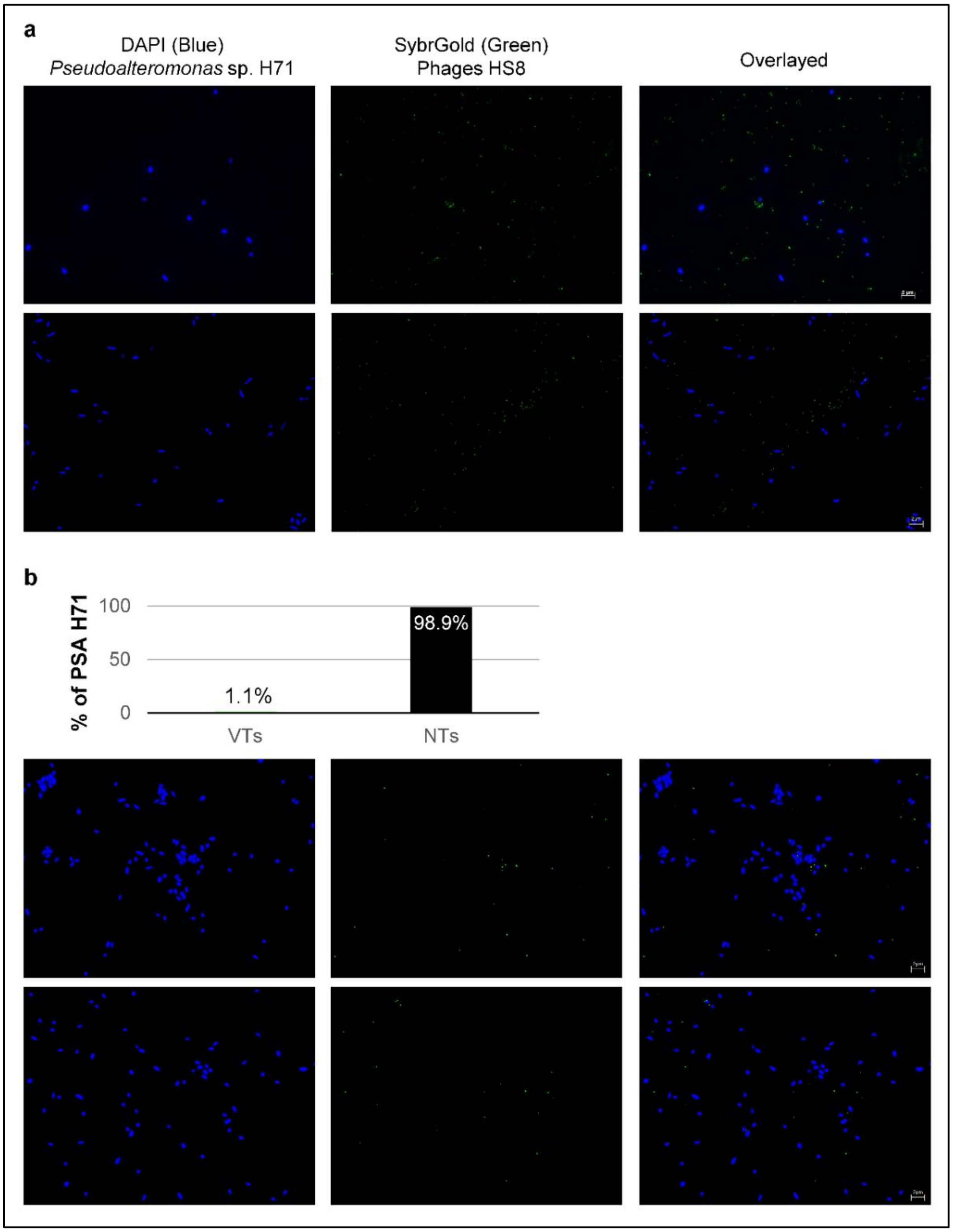

Fig S7. Microscopic evaluation of viral-tagging at MOI=10, but using a non-specific phage. **A** and **B**, Microscopic observation of virus-tagged cells (VTs, green-colored viral particles on the margin of blue-colored cells), nontagged cells (NTs, blue signals without green signals), and extracellular free phages (unbound green signals) from *Pseudoalteromonas* strain H71 cells infected by nonspecific phage HS8 at an MOI of 10. For the comparison of free phage removal, the samples were prepared without (A) and with (B) centrifugation and resuspension, respectively (see Methods). Representative microscopic images from a total of 545 cells were shown due to space limitations. Figure details as in Fig S4.

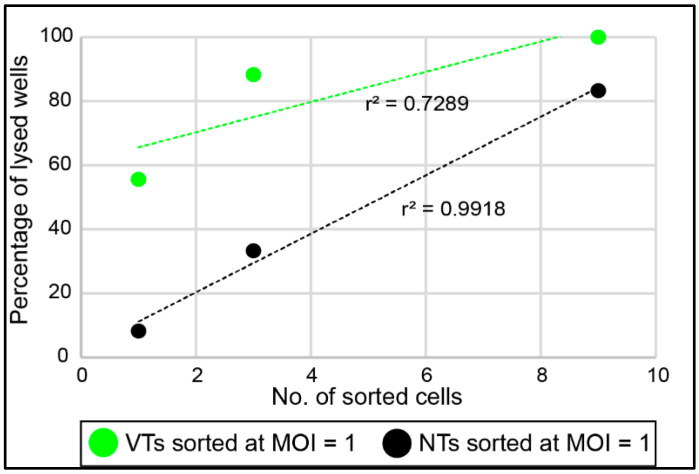

Fig S8. Linear regression relationships between the number of sorted cells and the percentage fraction of lysed wells. Correlations between the percentages of lysed wells (y-axis) and the numbers (x-axis, n = 1, 3, and 9) of virus-tagged (green) and nontagged cells (black) that were sorted from the infected cultures of *Pseudoalteromonas* strain H71 with phage HM1 at an MOI = 1. R-square values are represented.

Supplementary References

1. Duhaime MB, Solonenko N, Roux S, Verberkmoes NC, Wichels A, Sullivan MB. Comparative Omics and Trait Analyses of Marine Pseudoalteromonas Phages Advance the Phage OTU Concept. 2017;8(1241).
2. Wichels A, Biel SS, Gelderblom HR, Brinkhoff T, Muyzer G, Schütt C. Bacteriophage Diversity in the North Sea. Applied and Environmental Microbiology. 1998;64(11):4128.
3. Lara E, Holmfeldt K, Solonenko N, Sà EL, Ignacio-Espinoza JC, Cornejo-Castillo FM, et al. Life-Style and Genome Structure of Marine Pseudoalteromonas Siphovirus B8b Isolated from the Northwestern Mediterranean Sea. PLOS ONE. 2015;10(1):e0114829.
4. Kirzner S, Barak E, Lindell D. Variability in progeny production and virulence of cyanophages determined at the single-cell level. Environmental microbiology reports. 2016;8(5):605-13.
